# Subchondral insufficiency fracture of the medial femoral condyle treated conservatively with early non-weightbearing

**DOI:** 10.1016/j.radcr.2024.06.069

**Published:** 2024-07-18

**Authors:** Alexandros Maris, Rashed Al-Khudairi, Alexander Maslaris, Angelo V. Vasiliadis

**Affiliations:** a Department of Trauma and Orthopaedics, Royal Free Hospital NHS Trust, London NW3 2QG, United Kingdom; b Department for Health, University of Bath, Bath BA2 7AY, United Kingdom; c Radiology Department, Royal Free Hospital NHS Trust, London NW3 2QG, United Kingdom; d Department of Trauma and Orthopaedics, St. George's Hospital, London SW17 0QT, United Kingdom; e Department of Orthopaedic Surgery, St. Luke's Hospital, Thessaloniki 55236, Greece

**Keywords:** Spontaneous osteonecrosis of the knee, Early stage, Atraumatic knee pain, Therapy, Nonweightbearing

## Abstract

Spontaneous osteonecrosis of the knee (SONK) is a poorly understood but debilitating disease, that is a common cause of unilateral acute knee pain and swelling. The term “SONK” has been replaced by the term “subchondral insufficiency fracture” in the latest pathology and imaging literature. Few studies investigated the pathogenesis of SONK by examining the histological changes of the tissues. Very recently, the development of SONK was associated with a meniscal root tear. In terms of the preferred imaging, plain radiographs can confirm the diagnosis in late stages; however, magnetic resonance imaging (MRI) scan is often required. Regarding the treatment, conservative management is usually the treatment of choice in early stages, including a period of non-weightbearing or the use of medications, such as nonsteroidal anti-inflammatory drug (NSAIDS) or bisphosphonates. However, when SONK progresses, often a surgical intervention is required, such as knee replacement, but also minimally invasive techniques, such as arthroscopic intervention, have been described. We present a case of early SONK and discuss the possible pathogenesis of SONK, the clinical presentation, the radiological findings, and we focus on the importance of early diagnosis and early off-load period that is required to prevent further progression of the disease.

## Introduction

Spontaneous osteonecrosis of the knee (SONK), first described by Ahlbäck, Bauer et Bohne in 1968 [1] is a poorly understood but debilitating disease that affects mostly the medial femoral condyle, but also cases affecting the lateral femoral condyle, the patella and the proximal tibia have been reported in literature [2]. Since the precise cause of SONK was unknown, several theories, including those involving trauma and vascular insufficiency had been reported [3]. Nevertheless, it was never obvious why osteonecrosis might suddenly occur in the absence of any known risk factors. Only few studies investigated the pathogenesis of SONK by examining the histological changes of the tissues. In ten biopsy specimens of early-stage SONK lesions, Takeda et al. discovered a subchondral fracture devoid of any osteonecrotic lesion characteristics on early stages of SONK. In contrast, osteonecrotic lesions were present in advanced stages, and they were limited to the region distally to the fracture site. In these cases, the development of fibrous tissue and cartilage indicated a delayed union or nonunion [4]. Recently, few studies tried to investigate the effect of a meniscal root tear to the development of SONK, as the one by Kennedy, Strauss and LaPrade [5], where it was reported that meniscal root tears biomechanically interfere with normal joint loading and cause joint overload, which increases the risk of SONK and early arthritis. Additionally, Hashimoto et al. in 2021 [6] investigated the medial meniscus extrusion as an indicator of a bad prognosis for patients with SONK and they reported that patients with MRI finding of relative percentage of meniscal extrusion ≥ 33% are at high risk for progression of SONK. Additionally, few studies reported SONK after an anterior cruciate ligament reconstruction [7]. Most interestingly, a study by Malinowski et al. in 2022 [8], investigated the potential pathomechanisms through which a recent infection with SARS-CoV-2 may cause osteonecrosis of the knee, without the prior use of corticosteroids and found that there was widespread bone oedema throughout the whole femoral condyle as opposed to the more localized oedema that is usually centered mostly in the subchondral region in classic SONK. In that study, the repeat MRI scan after a 10-week period, reported normal findings, advocating towards a transient SONK associated with SARS-CoV-2 infection. Lastly, many studies linked the development of SONK in elderly people with low bone mineral density.

When SONK was first reported by Ahlback et al. in 1968, the diagnosis was mostly made using scintigraphy. Radiographs in early stages are unremarkable, but the classic region in the subchondral bone of the medial femoral condyle, with localized or diffuse T1 hypointense signal, and variable T2 signal are among the MRI characteristics of SONK that have been documented since 1988 [9], making the MRI scan the gold standard for confirming SONK. Interestingly, a very recent study by Yoo et al in 2022, proposed the use of single-photon emission computed tomography (SPECT CT) for early detection of SONK [10].

Regarding the management, the treatment for SONK can be either conservative or surgical and is dependent on the extent of the lesion and staging in accordance with the Koshino X-ray classification [11]. In early stages, most of the patients with SONK respond well to conservative management, which includes a period of non-weightbearing or the use of medications, such as NSAIDS or bisphosphonates [12]. Joint preserving measures should be taken into consideration if the patients are in the precollapse state. Osteochondral autograft might be helpful if patients advanced to subchondral collapse, although joint arthroplasty appears to be the preferred course of treatment, that's why it is crucial for the patient that the SONK is diagnosed early and immediate non-weightbearing is applied to prevent collapse of the femoral condyle.

We present a case of spontaneous osteonecrosis of the knee on a nonelderly patient with no predisposing risk factors, where a short nonweightbearing period resulted in significant improvement of the symptoms and prevented the patient from any surgical intervention.

## Case report

A 38-year-old female presented to the elective outpatient clinic with 1-month history of right knee pain and reduced range of motion. She had no history of trauma and no previous injury or pain on the knee. She had a past medical history of endometriosis, which was well controlled with paracetamol and NSAIDS and the patient has never been treated with oral or intraarticular corticosteroids. She worked an office-based job and exercised occasionally.

On inspection, the patient was mobilizing with an antalgic gait. On examination, there was no obvious deformity or swelling. There was a mild effusion on patellar tap and sweep test. Quadriceps bulk was symmetrical. There was no evidence of infection and the patient was systemically well. On palpation, she experienced pain over the medial femoral condyle, over the vastus medialis obliquus and over the medial joint line and the flexion of the knee above thirty degrees elicited significant pain over the medial aspect of the knee. The active range of motion was from full extension up to ninety degrees of flexion, while she could only tolerate an extra 10 degrees passively. The knee was ligamentously stable and Apley's, McMurray's and Thessaly's test elicited pain over the medial joint line.

**Fig. 1:**
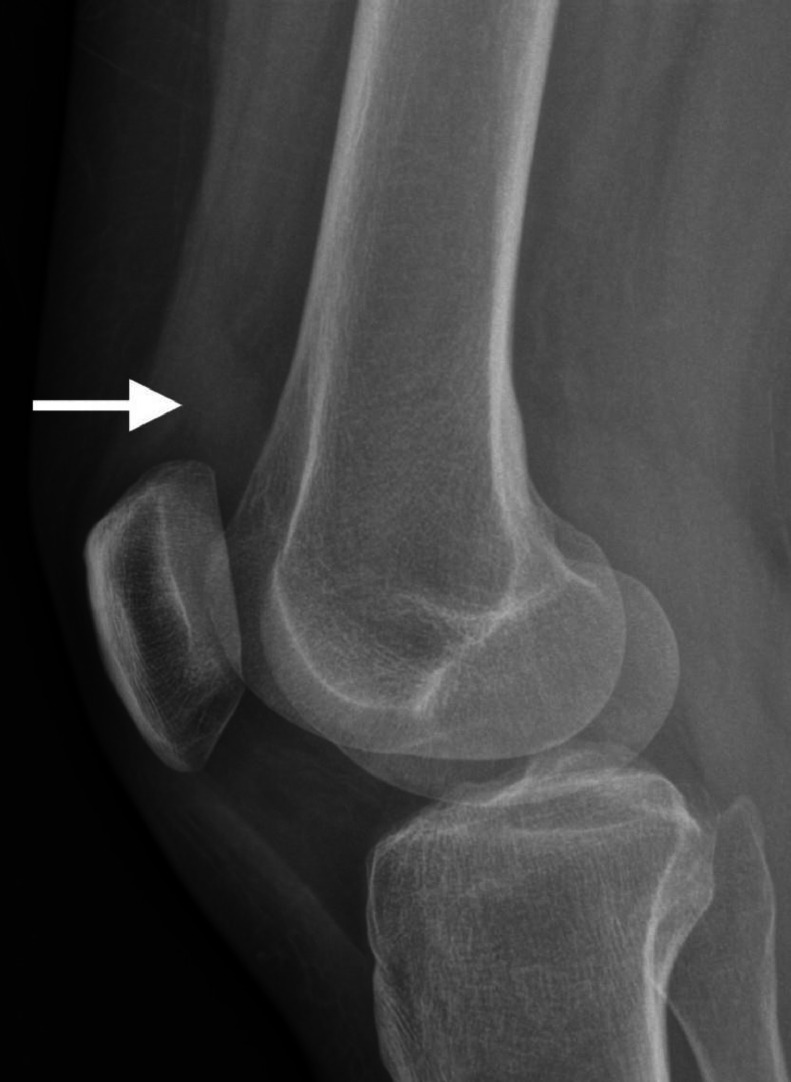
Lateral radiograph of the knee. There is a small suprapatellar knee effusion. No lipohaemarthrosis. No bony injury is identified.

Laboratory tests, including inflammatory markers and urate level were normal. The patient initially had X-rays (Fig. 1), which revealed no obvious bony injury but evidence of suprapatellar effusion. The suprapatellar effusion measured 11 mm on the lateral radiograph in anteroposterior dimension, which given it was greater than 10 mm, increases the likelihood of internal derangement according to Cecava et al. [13].

Due to the severity of symptoms, an MRI scan was booked to rule out an intra-articular pathology, including a medial meniscal tear.

The effusion measured a maximum anteroposterior diameter of 5 mm at the lateral patellofemoral recess on MRI.

**Fig. 2:**
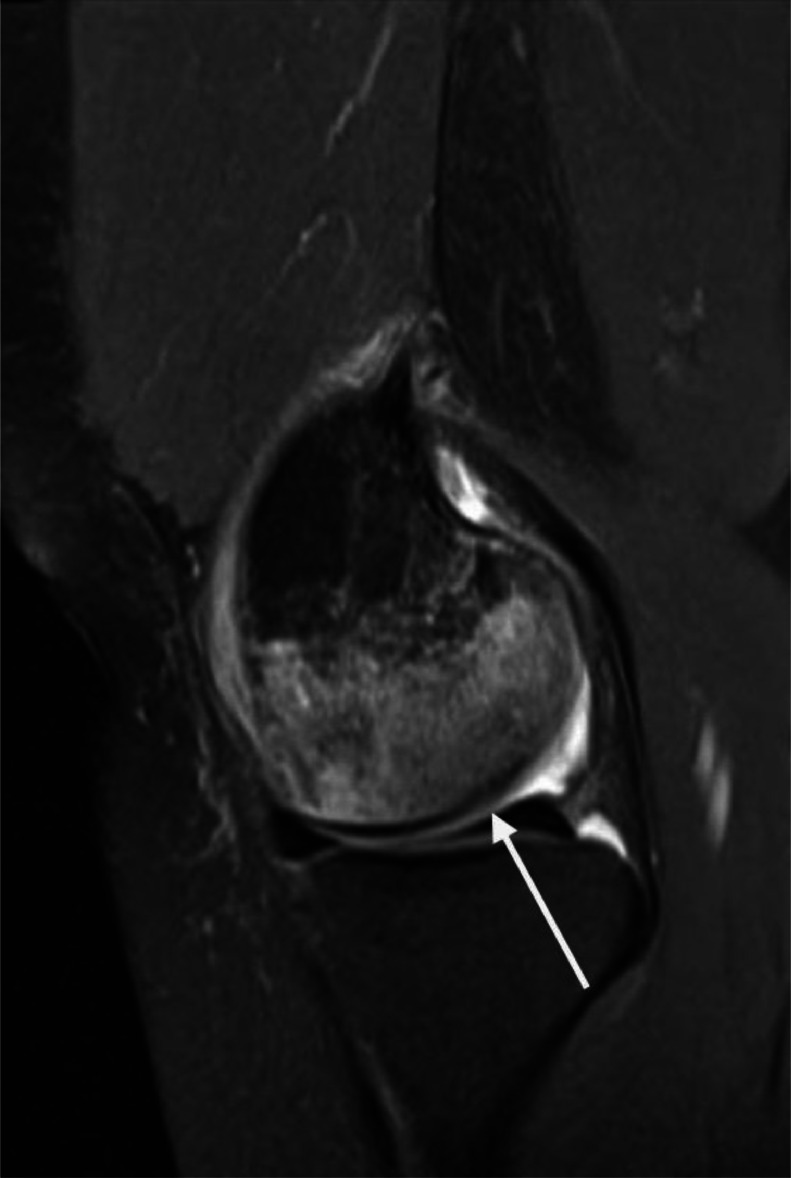
Sagittal PD fluid sensitive MRI sequence. There is a small knee effusion with intact menisci. There is florid high signal within the medial femoral condyle in keeping with extensive marrow oedema. There is irregularity of the subchondral bone plate.

**Fig. 3:**
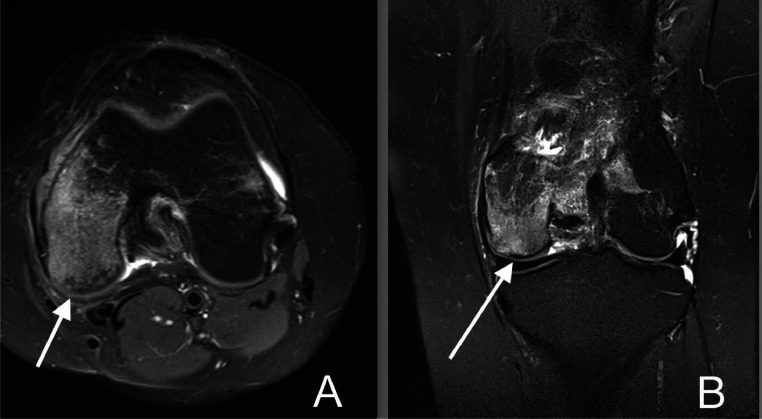
Axial (A) and coronal (B) PD fluid sensitive MRI sequences. There is indentation of the articular cartilage at the medial femoral condyle with irregularity at the subchondral bone plate, in keeping with chondral injury. There is intense oedema at the medial femoral condyle which appears out of proportion to the degree of chondral defect. Findings are suggestive of subchondral insufficiency fracture.

There was florid high proton density (PD) signal throughout the medial femoral condyle in keeping with marrow oedema (Fig. 2). There was also indentation of the articular cartilage at the medial femoral condyle involving a length of 16 mm in keeping with a chondral injury (Fig. 3). The oedema was out of proportion to the degree of chondral injury, raising the suspicion of SONK. The lateral compartment and patellofemoral compartment chondral surfaces were preserved with no significant arthropathy at the knee. No intra articular body was demonstrated.

Following the MRI findings, the patient was advised to off-load the knee for 4 weeks with crutches and a physiotherapy referral was made. When she presented to the clinic 6 weeks later, she had a normal gait, full range of movement and was pain free in keeping with complete resolution of symptoms.

## Discussion

SONK is a poorly understood disease entity, which if it is not diagnosed and treated early, can cause detrimental effects on patient's mobility and function, and can lead to further surgeries, with the associated risks. Recognition of early symptoms is crucial, and several studies proposed the use of MRI scan, when X-ray findings are unremarkable, especially for nonelderly patients. A subset of patients with early SONK, who initially have normal plain radiographs, but eventually develop irreversible osteonecrosis are described in the literature. Several studies attempted to associate the MRI findings with the prognosis. The presence of bone marrow oedema (BME) is associated with several conditions. The most crucial difference between early SONK and BME is the presence of a focal subchondral lesion on MRI, as studied by Yates et al. [14]. Lecouvet et al.'s [15] goal was to identify MRI prognostic markers for these early, pre–X-ray SONKs. A T2 subchondral signal of 14 mm in length or 4 mm in depth was always a bad prognostic characteristic in their series. A significant T2 signal was associated in 75% of instances by Bjorkengren et al. [16] with a bad clinical outcome, and in none of their cases with a favorable outcome. Lastly, Horikawa et al. [17] in 2016 studied the correlation between SONK, MRI scan and dual-energy X-ray absorptiometry (DEXA) scan and they concluded that the size of the afflicted lesion and decreased bone mineral density are closely related to the pathogenesis and prognosis of SONK.

Regarding the treatment, several studies exist in literature. In terms of the conservative management, non-weightbearing and/or knee extension brace have been proposed, but with no specific guidelines for early SONK. Bittner and Hartstein in 2018 [18] proposed a 4-week period of nonweightbearing with knee in full extension, as an adequate period for early SONK. Zhen et al. [19] conducted a protocol regarding the use of bisphosphonates as treatment for SONK, while Horikawa et al. [20] in 2020 trailed a daily dose of teriparatide and concluded that it can reduce the size of the affected SONK lesions. On the other hand, several surgical options have been studied. Jauregui et al. [21] conducted a meta-analysis of the studies about unicompartmental knee arthroplasty for SONK and concluded that it could be as effective as the total knee replacement, which is the most preferred choice of treatment for late stages. Other surgical options include high tibial osteotomy [22], knee arthroscopy for either diagnostic reasons or for addressing a possible meniscal root tear, proximal fibula osteotomy [23] and osteochondral allograft transplantation [24].

## Conclusion

Due to the disease's deceptive onset and vague symptoms, spontaneous osteonecrosis of the knee can be really difficult to diagnose and treat. Patients are more likely to develop severe osteoarthritis if the pathology is left undetected, especially in cases that are slow or silent. Thus, reducing this risk can be aided by early detection with the use of MRI scan and cautious nonoperative interventions, such as early nonweightbearing and/or knee extension brace, that can prevent the femoral condyle collapse. This is very important especially in nonelderly people, as even the surgical options, can have detrimental effects on their mobility and function. Early detection and full resolution of the symptoms with simple conservative measures can also prevent patients from the associated risks of the surgical alternatives. As far as we are aware, there are no specific guidelines for managing subchondral insufficiency fractures of the medial femoral condyle in early phases. We suggest that a 4-week non-weightbearing time is sufficient for treating these fractures. We are aware that this is only applicable to healthy, fit patients who do not have any risk factors.

## Patient consent

Verbal and written informed consent for the publication of this case report was obtained from the patient.
